# Modulation of Muscle Pain Is Not Somatotopically Restricted: An Experimental Model Using Concurrent Hypertonic-Normal Saline Infusions in Humans

**DOI:** 10.3389/fpain.2020.601544

**Published:** 2020-12-15

**Authors:** James S. Dunn, David A. Mahns, Saad S. Nagi

**Affiliations:** ^1^School of Medicine, Western Sydney University, Penrith, NSW, Australia; ^2^Department of Biomedical and Clinical Sciences, Center for Social and Affective Neuroscience, Linköping University, Linköping, Sweden

**Keywords:** normal saline, muscle afferent, somatotopy, muscle pain, hypertonic saline, hyperalgesia, central sensitization

## Abstract

We have previously shown that during muscle pain induced by infusion of hypertonic saline (HS), concurrent application of vibration and gentle brushing to overlying and adjacent skin regions increases the overall pain. In the current study, we focused on muscle-muscle interactions and tested whether HS-induced muscle pain can be modulated by innocuous/sub-perceptual stimulation of adjacent, contralateral, and remote muscles. Psychophysical observations were made in 23 healthy participants. HS (5%) was infused into a forearm muscle (flexor carpi ulnaris) to produce a stable baseline pain. In separate experiments, in each of the three test locations (*n* = 10 per site)—ipsilateral hand (abductor digiti minimi), contralateral forearm (flexor carpi ulnaris), and contralateral leg (tibialis anterior)—50 μl of 0.9% normal saline (NS) was infused (in triplicate) before, during, and upon cessation of HS-induced muscle pain in the forearm. In the absence of background pain, the infusion of NS was imperceptible to all participants. In the presence of HS-induced pain in the forearm, the concurrent infusion of NS into the ipsilateral hand, contralateral forearm, and contralateral leg increased the overall pain by 16, 12, and 15%, respectively. These effects were significant, reproducible, and time-locked to NS infusions. Further, the NS-evoked increase in pain was almost always ascribed to the forearm where HS was infused with no discernible percept attributed to the sites of NS infusion. Based on these observations, we conclude that intramuscular infusion of HS results in muscle hyperalgesia to sub-perceptual stimulation of muscle afferents in a somatotopically unrestricted manner, indicating the involvement of a central (likely supra-spinal) mechanism.

## Introduction

For most individuals, it is relatively easy to distinguish between innocuous and noxious stimuli. However, in a subset of individuals afflicted with chronic pain, there is a disturbance of normal somatosensory function, such that a normally innocuous stimulus can evoke pain, for example, the emergence of tactile allodynia in patients with sciatica (1). This can have a debilitating impact on both the individual and society (2, 3).

Studies using hypertonic saline (HS) infusions have shown a touch-evoked pain (allodynia) that extends to overlying (4) and adjacent (5, 6) skin regions. Intramuscular HS administration produces a deep musculoskeletal pain that often extends or refers to distal regions (7–9). Repeated intramuscular injections of HS reveal plastic processes with a decrease in the area and intensity of local pain and an increase in the expression of referred pain (10) in addition to the emergence of pain hypersensitivity that extends bilaterally (11). These complex interactions cannot readily be explained by changes in peripheral circuitry and appear to mimic characteristics of chronic pain conditions such as fibromyalgia. Within such chronic pain conditions, current arguments favor an explanation based on a central change in, or sensitization of, the neural function that results in the observed widespread and diffuse musculoskeletal pain, pressure-pain hypersensitivity, cutaneous allodynia, and tactile dysesthesia (12–14).

In the current study, a HS infusion model was used to examine whether the interaction previously observed between muscle and skin (4, 6, 11) can be replicated between adjacent and remote muscles. We hypothesized that the presence of background nociceptive activity using HS infusion would produce a state of central sensitization resulting in an exacerbation of the overall pain (hyperalgesia) to the application of a normally innocuous stimulus (normal saline, NS). We also hypothesized that this effect would occur regardless of whether the NS was infused into an adjacent or a remote muscle.

## Methods

Twenty-three healthy naïve participants aged 18–28 years (six females), with no reported history of musculoskeletal or neurological disorders, were recruited for this study. Participants were asked to abstain from intensive bouts of exercise for 48 h preceding the experiment so as not to sensitize the target muscles (15). Six participants took part in multiple arms of the study across different experimental sittings (30 experiments total), the inclusion of these participants in multiple study arms was random. One participant took part in all arms of the study, whilst a further five participated in both the contralateral and remote testing procedures. To minimize the risk of a placebo effect or familiarization with the protocol, repeat participants did not undertake experiments in any prescribed order with control recordings in the absence of HS-infusion (i.e., no-pain) obtained at the commencement of each separate experiment session across each of the test locations.

Informed written consent was obtained from each participant prior to the experiment. This study was approved by the Human Research Ethics Committee (approval numbers: H9190 and H13204) of Western Sydney University in accordance with the revised Declaration of Helsinki.

Participants were comfortably seated in a chair throughout the experiment. HS and NS were infused using a Syringe Infusion Pump (Harvard Apparatus, South Natick, Massachusetts, USA) and a 25G winged infusion set. Importantly, the Syringe Infusion Pump used for NS-infusion was obscured from sight and did not include any audible cues. Pain ratings were continuously recorded using the ADInstruments Response Meter connected to the ADInstruments PowerLab (ADInstruments, Dunedin, New Zealand). The Response Meter had a slide control, and the pain scale was divided into ten equal segments within a range of 0 (no pain) to 10 (worst pain). In addition, participants were asked to verbally report the location of pain during the course of the experiment.

### Infusion of Hypertonic Saline

Across all parts of the study, 5% HS was infused into the belly of the flexor carpi ulnaris (FCU) muscle of the forearm for ~10 min to establish a stable baseline pain. The muscle belly was palpated whilst the participant performed light flexion and adduction of the wrist to identify the boundaries of the FCU muscle. The needle was inserted ~0.8–1 cm into the center of the muscle belly at an angle perpendicular to the skin at the site of insertion. The infusion rate of HS in the FCU varied between subjects (30–175 μl/min) to establish a moderate pain intensity preferably between 4 and 6 (out of 10) on the pain scale. Once a stable baseline pain was achieved, no further changes were made to the infusion rate.

### Infusion of Normal Saline

After a stable baseline pain was maintained for at least a minute, NS (0.9%) at room temperature was concurrently infused at the rate of 50 μl/min for 1 min per trial (tested in triplicate). This duration was chosen based on the data collected in a pilot study which indicated a delay of several seconds before the onset of an increase in pain levels. The delayed response has been reported in previous studies (6, 16). The triplicate NS trials were performed at 1-min intervals.

Participants were asked to continuously rate the overall pain intensity, and any changes thereof, on the pain scale. Care was taken to avoid the use of suggestive language with participants informed that the HS-induced pain could remain the same, increase or decrease during the co-infusion with NS.

In addition to concurrent HS-NS infusions, NS alone was infused in triplicate trials prior to the commencement and upon cessation of HS-evoked pain in all experiments. Typically, the HS-evoked pain disappeared over a time course of under 10 min. After a 3- to 5-min wait following cessation of pain, NS infusion was repeated at each site. Collectively, ~450 μl of NS was infused per muscle.

### Part 1: Interactions With Adjacent Muscles

NS was infused into the ipsilateral abductor digiti minimi (ADM) muscle of the hand to examine potential interactions between adjacent muscles in response to HS-induced acute muscular pain. The muscle belly of the ADM was identified by palpation whilst the participant abducted the fifth digit of their hand. The infusion needle was inserted to a depth of ~0.5 cm into the center of the ADM muscle belly. The ADM muscle was chosen as it shares the same peripheral innervation (ulnar nerve) as the HS-infused FCU.

### Part 2: Contralateral Interactions

NS was infused into the belly of the contralateral FCU muscle of the forearm to test whether the HS-NS interactions were limited to muscles within the same nerve territory or spread to contralateral muscles as well. The needle location and insertion for the contralateral FCU were identical to the HS-infusion site described prior.

### Part 3: Remote Interactions

NS was delivered to the belly of the tibialis anterior (TA) muscle of the contralateral leg to determine the spatial extent of inter-muscle interactions in an acute pain state. The muscle belly of the TA was identified by palpation during dorsiflexion of the ankle. The needle was inserted into the middle of the belly of the TA muscle perpendicular to the skin to a depth of ~1 cm.

### Statistical Analysis

Repeated measures two-way analysis of variance (RM 2-way ANOVA) was used to compare pain ratings at baseline (HS infusion alone) with evoked responses (co-infusion of NS and HS) at each location (adjacent, contralateral, and remote). Where a significant change (*P* < 0.05) was found, individual comparisons were made using Tukey's multiple comparison test. The normal distribution of data was confirmed in all groups using D'Agostino and Pearson omnibus normality test. Pain scores for the baseline (HS) and co-infusion (HS and NS) conditions are presented as mean ± standard error of the mean (SEM) for all parts of the study. Statistical analysis was performed using GraphPad Prism (version 7.04, La Jolla, California, USA).

## Results

**Figure 1:**
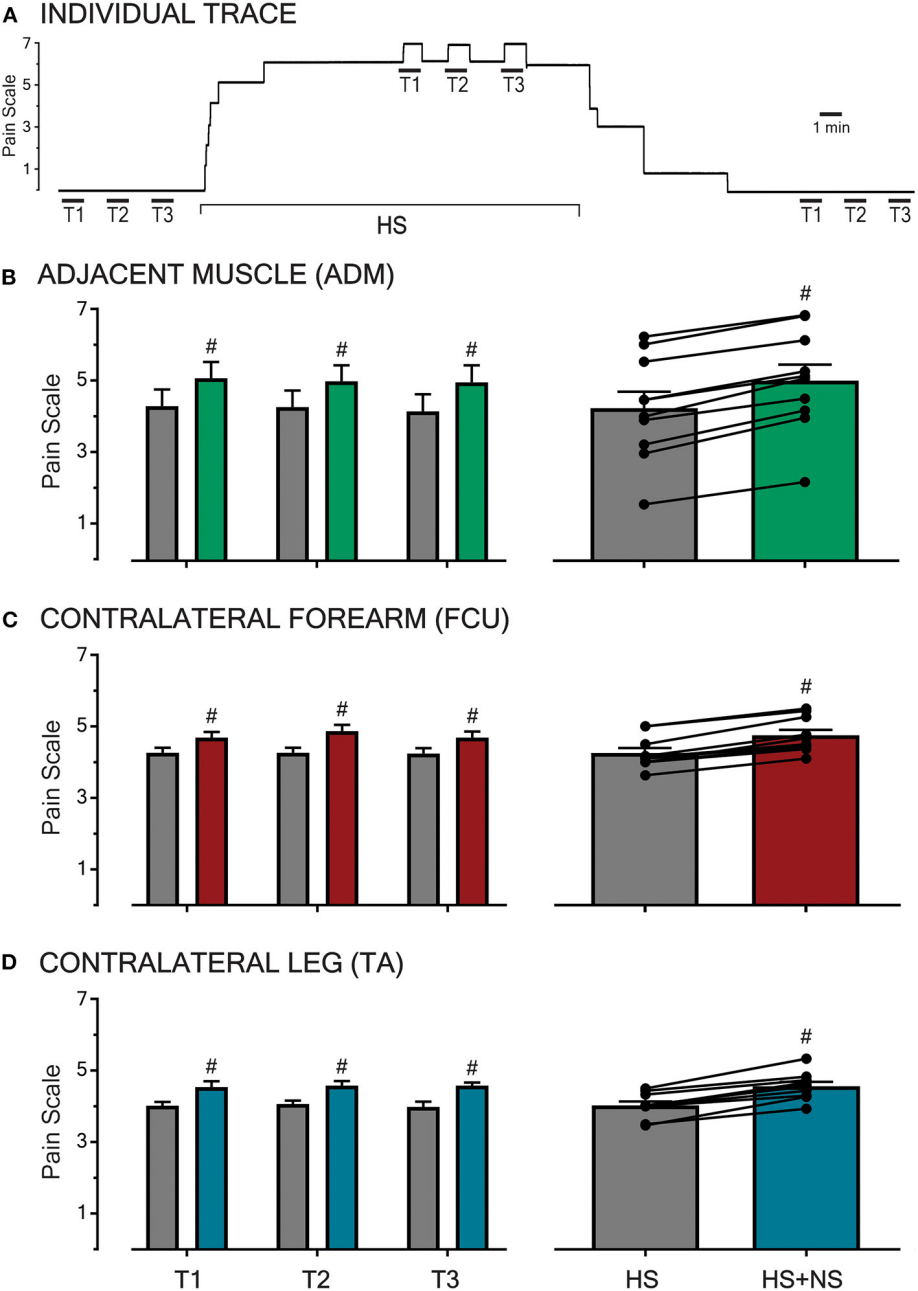
Pain intensities in response to HS-infusion and subsequent to transient NS-infusions at various sites across the body. An example raw trace of a participant's pain ratings throughout an experimental sitting is shown **(A)**. In the absence of background pain, infusions of NS for 1 min (T1, T2, T3 with infusion time-course shown by the overlying bar) were imperceptible. During baseline HS-induced muscle pain in the FCU, co-infusion of NS (triplicate, left **B–D)** produced a reproducible increase in overall pain. Following the cessation of HS infusion and the associated background pain (0 out of 10 on the pain scale), NS trials were once again imperceptible. In all three sessions, HS pain was generated in the FCU, and the test location for NS infusion was the adjacent ADM muscle **(B)**, the contralateral FCU **(C)**, or the contralateral TA muscle (**D**, remote). At each test location, NS co-infusion during HS background pain resulted in a reproducible and significant increase in overall pain (*P* < 0.0001, right **B–D**). The transient pain increase was reproducible across trials at all sites. Significant changes (*P* < 0.0001, #) were confirmed between baseline (HS) and co-infusion (HS + NS) using RM 2-way ANOVA.

Prior to the induction and following the cessation of HS-evoked muscle pain, all participants reported NS infusion (50 μl/min) to be innocuous (i.e., rated as 0 out of 10 on the pain scale) and imperceptible regardless of the NS infusion site (Figure 1A). The infusion of 5% HS into the FCU always resulted in a diffuse, deep pain in the muscle that extended down the medial aspect of the forearm. This baseline pain remained stable in the absence of NS co-infusions (Figure 1A) and did not significantly differ between the different parts of the study (*P* = 0.66).

At all three test locations (adjacent, contralateral, and remote), the co-infusion of NS significantly increased the overall pain in all trials (T1-3, *P* < 0.0001, Figures 1B–D left-hand panel). All observed increases in pain scores during co-infusion were transient and time-locked to the NS-infusion, with the pain returning to baseline (HS) within 1 min of the cessation of NS co-infusion (example shown in Figure 1A). Further, the increases in pain scores did not vary in amplitude based on the location (adjacent, contralateral, and remote) of the NS co-infusion (*P* = 0.30).

The pooled mean response of all participants in each part of the study, with respective HS and HS + NS data points linked, are shown in the right-hand panel of Figures 1B–D and described further in the following sections.

### Part 1: Interactions With Adjacent Muscles

The infusion of HS into the FCU resulted in a pooled mean score of 4.3 ± 0.5 (*n* = 10). When NS was co-infused into the adjacent ADM in the presence of this background pain, the pooled mean score increased to 5.0 ± 0.4 (Figure 1B). This constitutes a pain increase of ~16% and when comparing baseline and co-infusion pain scores the increase in pain ratings was significant [*P* < 0.0001, *F*_(1,27)_ = 318.5]. This indicates that muscle pain can be modulated by low-threshold/sub-perceptual stimulation of an adjacent muscle.

### Part 2: Contralateral Interactions

The infusion of HS into the FCU resulted in a pooled mean score of 4.3 ± 0.1 (*n* = 10). The co-infusion of NS into the contralateral FCU increased this pooled mean score to 4.8 ± 0.2 (Figure 1C). This represents a ~12% increase in the pain scores during co-infusion, an effect found to be significant [*P* < 0.0001, *F*_(1,27)_ = 156.7]. This demonstrates that muscle pain can be modulated by normally sub-perceptual stimulation across contralateral muscles, thereby suggesting a central (spinal/supra-spinal) phenomenon.

### Part 3: Remote Interactions

Within this aspect of the study, participants reported a pooled mean score of 4.0 ± 0.1 in response to infusions of HS into the FCU (*n* = 10). During concomitant infusion of NS into the contralateral TA, participants reported a pain increase of 15% with the pooled mean score increasing to 4.6 ± 0.1 (Figure 1D). A comparison of the baseline and co-infusion pain scores revealed a significant difference [*P* < 0.0001, *F*_(1,27)_ = 97.84]. The observed interaction between the site of noxious muscle stimulation and remote innocuous muscle stimulation alludes to the involvement of a supra-spinal mechanism.

**Figure 2:**
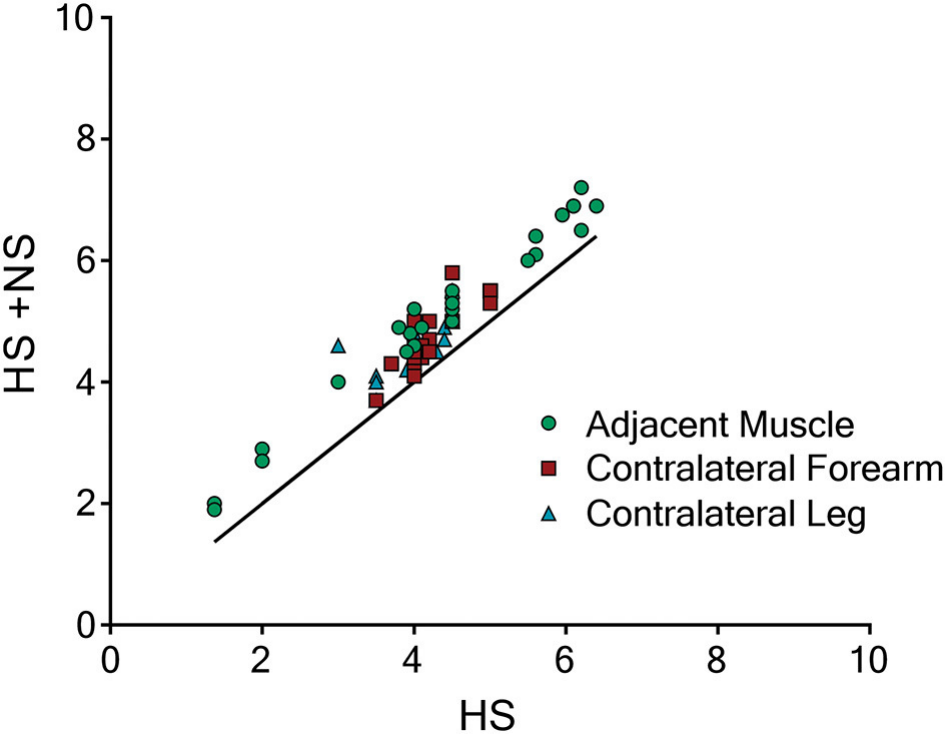
Triplicate data points for each participant during HS-NS co-infusion plotted as a function of baseline pain. When triplicate responses from each participant at each location (*n* = 10 per location, total *n* = 90) to NS infusion during HS infusion (HS + NS) are plotted as a function of baseline pain (i.e., HS alone) all data points fall to the right of the line of equivalence (x = y or HS = HS + NS). This indicates that the NS infusion evoked a reproducible effect between trials and across all test sites and over a broad range of baseline pain levels.

In Figure 2, triplicate responses for each individual (*n* = 10 per test location) at all three test sites (*n* = 90) to transient NS infusion during HS infusion (i.e., HS + NS) have been plotted as a function of the baseline pain evoked by HS alone. When plotted in this manner, all data points fell to the left of the line of equivalence (x = y or HS = HS + NS) indicating that the NS infusion evoked a reproducible pain increase across a broad range (pain scale 1.4–6.7) of baseline pain levels.

When participants were asked about the location of pain, all participants—except 2 in part 1 and one each in parts 2–3—ascribed it to the forearm where hypertonic saline was infused with no discernible percept attributed to the sites of NS infusion. This was true not only for HS-evoked pain but also for pain increases during HS-NS co-infusions. The four subjects who did not ascribe the pain increase to the HS-infusion site instead ascribed it to the NS-infusion site. Importantly, these subjects always reported NS-infusion as imperceptible at the local site under control and recovery conditions (no HS-pain). This suggests that NS infusion was almost always nonpainful regardless of whether there was HS pain or not, but in the presence of HS pain, the NS co-infusion resulted in hyperalgesia at the HS site, and this modulation of HS pain was not somatotopically restricted.

## Discussion

The current study has provided evidence that muscle pain can be modulated (hyperalgesia) by sub-perceptual stimulation of muscle afferents in a somatotopically unrestricted manner. This finding not only builds upon the previous observation that an intramuscular HS infusion can result in allodynia in the overlying and adjacent skin regions (4, 6, 17) but the spatial extent of this modulation, spanning several spinal segments, suggests the involvement of a central, likely supra-spinal, mechanism.

The sub-perceptual nature of repeated intermittent NS infusions (50 μl over 1 min) under control (no HS-pain) condition suggests that localized muscle distension does not activate the nociceptors (18) but may activate low-threshold stretch-sensitive receptors within the muscle. In this respect, these weak mechanical stimuli resemble the inability of weak (micro) intraneural electrical stimulation to produce a discernible pain sensation at recording sites dominated by muscle spindles (19, 20). We have also previously shown that intradermal infusions of NS (50 μl/min for 2 min) do not produce a percept (5).

The conversion of the sub-perceptual NS stimulus to one that enhances pain, during HS infusion in the FCU muscle, is unlikely to be due to peripheral sensitization given the anatomical separation (forearm vs. hand, >15 cm) and the small volume of intermittently infused NS. Likewise, the increase in pain evoked by NS-infusion into the contralateral forearm is more consistent with a central involvement. Furthermore, the interaction between the FCU and the contralateral TA suggests that the central involvement likely extends to supra-spinal structures. Assertions as to the exact location of this central involvement cannot be resolved by this study, but the acute/short-lasting and reversible nature of these interactions do suggest that the requisite circuitry may already be present, and thus an elaborate anatomical reorganization need not be necessary for these to occur.

The broad-ranging muscle-muscle interactions observed here appear to be in marked contrast to the somatotopically constrained interactions observed in the skin; for example, the confinement of secondary hyperalgesia to the region immediately surrounding intradermal capsaicin injection (21–23) or the inability of microstimulation of large-diameter mechanoreceptors innervating a skin region beyond the site of secondary hyperalgesia to produce a painful percept (16).

The effects observed in the current study are most likely driven by a transient and reversible episode of central sensitization (increased excitability and synaptic efficacy of central nociceptive pathways) (24) in response to the HS-induced muscle pain. The HS infusion alone was run for ~10 min prior to the commencement of NS co-infusion, and this may have resulted in a state of central sensitization. Indeed, the clinical correlates of central sensitization (25, 26) are apparent in a HS-infusion model with hyperalgesia and allodynia reported in this and previous work (4, 6, 11, 17).

The generalized modulation of the exacerbated pain response at the HS-induced muscle site during NS-infusion in the adjacent, contralateral, and remote muscles is noteworthy and warrants further study using more quantitative measures of pain localization than verbal reporting. Further, the quality and temporal characteristics of this hyperalgesia need further investigation. In addition to the prerequisite of ongoing nociceptive input (HS infusion), we observed that the onset of NS-evoked increase in pain tended to be delayed by several seconds, which suggests a possible need for temporal summation.

Previous findings in humans have shown that repeated intramuscular HS injections in the TA result in a pressure-pain hypersensitivity developing across both the ipsilateral and contralateral TA muscles (11). Further, it has been shown in animals that a unilateral forelimb injury can produce sensory perturbations in the contralateral limb (27). In the case of intramuscular HS, the evidence for centralized effects necessitates the need for control data collection prior to any HS administration and warrants an investigation into other commonly used pain models.

In the current study, the co-infusion of sub-perceptual NS resulted in increased HS-pain (i.e., hyperalgesia). In HS and other experimental models as well as chronic pain conditions, tactile and thermal stimuli can produce allodynia (pain to a normally nonpainful stimulus) and hyperalgesia (increased pain from a painful stimulus) (1, 4, 6), but paradoxically, these modulatory stimuli—both painful and nonpainful—can also reduce pain with slow gentle brushing of the skin shown to reduce cutaneous heat pain (28). Conditioned pain modulation is a well-recognized phenomenon in which a painful stimulus can be inhibited by a second painful stimulus applied to a different body site (i.e., pain inhibits pain) (29–31). The underlying mechanisms are not fully understood but likely involve a complex interplay between excitatory and inhibitory circuits in the central nervous system.

## Data Availability Statement

The raw data supporting the conclusions of this article will be made available by the authors, without undue reservation.

## Ethics Statement

The studies involving human participants were reviewed and approved by Human Research Ethics Committee of Western Sydney University. The patients/participants provided their written informed consent to participate in this study.

## Author Contributions

SN and DM contributed to the conception and design of the study. JD performed the experiments and organized the database and wrote the first draft of the manuscript. All authors contributed to the article and approved the submitted version.

## Conflict of Interest

The authors declare that the research was conducted in the absence of any commercial or financial relationships that could be construed as a potential conflict of interest.
